# P073. Impaired oxidative balance in migraine: an open study

**DOI:** 10.1186/1129-2377-16-S1-A115

**Published:** 2015-09-28

**Authors:** Vincenzo Pizza, Domenico Cassano, Vincenzo Busillo, Anella Agresta, Eugenio Luigi Iorio

**Affiliations:** Neurophysiopatology Unit, S. Luca Hospital, ASL Salerno, Vallo della Lucania, SA Italy; Headache Centre, District N. 60, ASL Salerno, Nocera Inferiore, SA Italy; Neurology Unit, S. Maria della Speranza Hospital, ASL Salerno, Eboli, SA Italy; International Oxidative Stress Observatory, Salerno, Italy

## Introduction

Migraine is the most common neurological disorder, but the molecular basis is still not completely understood. An impairment of mitochondrial oxidative metabolism might play a role in the pathophysiology. Moreover there is strong evidence associating migraine with a variety of comorbid disorders, including cardiovascular disease and stroke, in which oxidative stress seems to be an important underlying mechanism. However, data are in part controversial and the possible underlying mechanism remains elusive to date. Also, the data regarding the interictal state in migraineurs is limited.

## Objective

Aim of this study was to evaluate the oxidative balance in a sample of patients with migraine by means of routine specific serum tests, such as d-ROMs test and BAP test.

## Materials and methods

One hundred outpatients, (74 F, 26 M), mean age 39.2 years (SD 13.2), range 18-62 years, suffering from migraine without aura (ICDH-II 2004 criteria) were enrolled. The mean duration of disease was 1.8 (SD 0.8) years, range 1-3 years. Serum total oxidant capacity was determined by performing the d-ROMs test, whose chemical principle is based on the ability of a biological sample to oxidize N,N-diethylparaphenylenediamine (normal range 250-300 CARR U, where 1 CARR U is equivalent to 0.8 mg/L H2O2), while serum total antioxidant capacity was assessed by means of BAP test, which measures the ability of a serum sample to reduce iron from the ferric to the ferrous ionic form (optimal value >2200 micromol/L reduced iron).

## Results

Mean values of d-ROMs tests were 397.5 CARR U (SD 144.3) while mean values of BAP test were 1758.2 micromol/L reduced iron (SD 485.7).

According to the data, enrolled patients were found to be in a classical condition of oxidative stress. In fact, compared to the normal range, oxidant capacity, as measured by means of d-ROMs test, was increased (>300 CARR U) and biological antioxidant potential (as measured by means of BAP test) was decreased (<2200 micromol/L reduced iron).

## Conclusions

Although preliminary, our study confirms that migraine without aura is associated to oxidative stress and suggests that d-ROMs test and BAP test can be useful to identify an oxidative unbalance in clinical routine of patients suffering from this frequent disease. Our data suggest that oxidative stress may represent a key event in the pathophysiology of migraine and a suitable therapeutic target. Further knowledge about this issue may contribute to understanding the cause and complications of migraine and may be essential for development of treatment approaches.

Written informed consent to publication was obtained from the patient(s).

